# A parasitic coevolution since the Miocene revealed by phase-contrast synchrotron X-ray microtomography and the study of natural history collections

**DOI:** 10.1038/s41598-020-79481-x

**Published:** 2021-01-29

**Authors:** Michel Perreau, Danny Haelewaters, Paul Tafforeau

**Affiliations:** 1 Université de Paris, IUT Paris Pajol, Paris, France; 2 Department of Organismic and Evolutionary Biology, Harvard University, Cambridge, MA USA; 3 Department of Zoology, University of South Bohemia, České Budějovice, Czech Republic; 4 Department of Botany and Plant Pathology, College of Agriculture, Purdue University, West Lafayette, IN USA; 5 European Synchrotron Radiation Facility, Grenoble, France

**Keywords:** Zoology, Coevolution

## Abstract

The discovery of a new fossil species of the Caribbeo-Mexican genus *Proptomaphaginus* (Coleoptera, Leiodidae, Cholevinae) from Dominican amber, associated with a new fossil parasitic fungus in the genus *Columnomyces* (Ascomycota, Laboulbeniales), triggered an investigation of extant species of *Proptomaphaginus* and revealed the long-enduring parasitic association between these two genera. This effort resulted in the description of the fossil species †*Proptomaphaginus alleni* sp. nov., and one fossil and two extant species of *Columnomyces*, selectively associated with species of *Proptomaphaginus*: †*Columnomyces electri* sp. nov. associated with the fossil †*Proptomaphaginus alleni* in Dominican amber, *Columnomyces hispaniolensis* sp. nov. with the extant *Proptomaphaginus hispaniolensis* (endemic of Hispaniola), and *Columnomyces peckii* sp. nov. with the extant *Proptomaphaginus puertoricensis* (endemic of Puerto Rico). Based on biogeography, our current understanding is that the Caribbean species of *Proptomaphaginus* and their parasitic species of *Columnomyces* have coevolved since the Miocene. This is the first occurrence of such a coevolution between a genus of parasitic fungus and a genus of Coleoptera. The phylogenetic relations among *Proptomaphaginus* species are also addressed based on a parsimony analysis. Fossil specimens were observed by propagation phase-contrast synchrotron X-ray microtomography (PPC-SRμCT) and extant specimens were obtained through the study of preserved dried, pinned insects, attesting for the importance of (i) technological advancement and (ii) natural history collections in the study of microparasitic relationships.

## Introduction

There is scientific consensus for the idea that the number of fungal parasites is highly underestimated^1–6^. Only 1.5% of insect-associated fungi are thought to be currently known^5^. These include necrotrophic and biotrophic parasites^7^. Necrotrophs kill their hosts and use dead host cells as a source for nutrition. Well-known examples are found in the Hypocreales (Ascomycota, Sordariomycetes), e.g., in the genera *Beauveria*, *Cordyceps*, *Ophiocordyceps*, and *Tolypocladium*, among others. Biotrophic parasites require a living host. Known groups of fungal biotrophs are the Asellariales and Harpellales (Zoopagomycota, Kickxellomycotina; formerly known as trichomycete fungi), Herpomycetales and Laboulbeniales (Ascomycota, Laboulbeniomycetes), and Septobasidiales (Basidiomycota, Uredinomycetes)^7–9^. Certain phytopathogens can be hemibiotrophic parasites—they require a living host, which is killed at later stages of infection. An example of this type of parasitism is *Magnaporthe grisea* (Sordariomycetes, Magnaporthales). In this paper, we will focus on the Laboulbeniales, one of three orders of arthropod-associated fungi in the Laboulbeniomycetes^10,11^.

Arthropod–Laboulbeniales associations are vastly understudied^12,13^, and fossil records of such associations are particularly scarce and poorly documented. Only one fossil species has been reported in the literature. This is †*Stigmatomyces succini*, a parasite of the fly †*Prosphyracephala succini* (Diptera, Diopsidae)^14^. It was discovered in a fragment of Bitterfeld amber in central Germany, which at that time was described as Baltic amber redeposited in the Miocene^14,15^. The age of the amber-bearing layer is uppermost Oligocene. More specifically, the amber piece was removed from the “Bernsteinschluff” Horizon in the upper part of the Cottbus Formation at the Goitsche mine near the city of Bitterfeld. Biostratigraphic data point at an uppermost Chattian age for this amber-bearing sediment, around 25.3–23.8 million years^16,17^, whereas Baltic amber is usually considered at least 35 million years old^18^.

The hosts of extant species of Laboulbeniales are representatives of Arthropoda. Primarily beetles, but a variety of other insects (ants, flies, cockroaches) and also millipedes, harvestmen, and mites can serve as hosts. As an exception among multicellular fungi, species of Laboulbeniales exhibit determinate growth; the fungus develops by regulated mitotic divisions resulting in a single *thallus* with a defined number of cells. Among Laboulbeniales, the genera *Columnomyces*, *Euzodiomyces*, *Kainomyces*, *Scepastocarpus*, and *Zodiomyces* are unique in forming relatively large thalli with many-celled pseudoparenchymatous receptacles, each with a single ascospore-forming organ (perithecium) or multiple perithecia^19,20^.

Extant species of Ptomaphagini (Coleoptera, Leiodidae, Cholevinae) are reported to host representatives of three genera of Laboulbeniales: *Columnomyces* on a Nearctic species of *Ptomaphagus*^19^; *Diphymyces* on species of *Adelopsis*^21,22^, *Ptomaphagus*^22,23^, and *Ptomaphaginus*^24^; and *Rodaucea* on species of *Adelopsis*^22,25^. Thus far, no Laboulbeniales had ever been reported for species of *Proptomaphaginus*.

The geographical distribution of the genus *Proptomaphaginus* (Cholevinae, Ptomaphagini, Ptomaphaginina) currently extends over Mexico and the West Indies, in a narrow latitudinal strip between 17° 25′ N and 23° 25′ N and from West to East in Mexico (two species), Cuba (two species), Hispaniola (one species), and Puerto Rico (one species)^26^. In addition, an undescribed species has been reported from the Bahamas^27^, extending the northern latitudinal limit of the distribution area of the genus to 25° N.

Currently, the genus *Columnomyces* contains a single species: *Columnomyces ptomaphagi*, found on a species of the genus *Ptomaphagus*. The present description of the first fossil association of the laboulbenialean genus *Columnomyces* with the coleopteran genus *Proptomaphaginus* in Dominican amber has led us to investigate extant species of *Proptomaphaginus*, which revealed the following one-to-one specific associations: †*Columnomyces electri* sp. nov.—†*Proptomaphaginus alleni* sp. nov. (fossil in dominican amber); *Columnomyces hispaniolensis* sp. nov.—*Proptomaphaginus hispaniolensis* Peck (in Hispaniola); *Columnomyces peckii* sp. nov.—*Proptomaphaginus puertoricensis* Peck (in Puerto Rico). The discovery of such a covolution—taken in the weak meaning of a one-to-one correspondence between parasite and host species, not in the strict sense because no cophylogeny was constructed—since the Miocene demonstrates an evolutionary long-lasting common history.

## Material and methods

### Material

**Figure 1:**
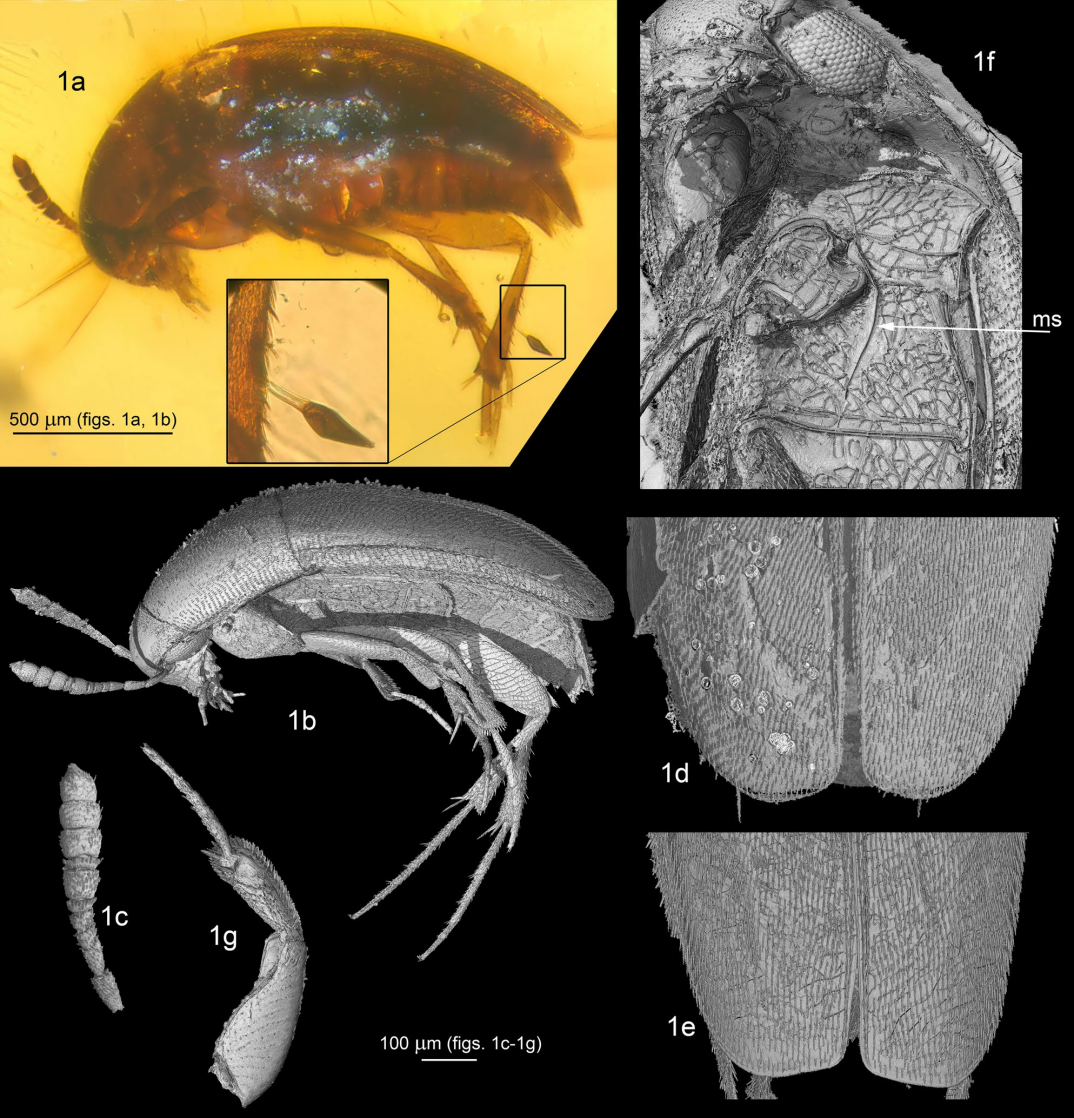
†*Proptomaphaginus alleni* sp. nov., external morphology. (**a**) paratype female, habitus lateral view with †*Columnomyces electri*. (**b**) holotype, lateral view (tomographic picture). (**c**) Antenna. (**d**) apex of elytra of female. (**e**) apex of elytra of male. (**f**) ventral structures. (**g**) foreleg. Abbreviation: ms = metaventral lateral suture.

Four specimens of †*Proptomaphaginus alleni* were examined. Two samples labelled as MP025 (male) and MP033 (female) are preserved at the Staatliches Museum für Naturkunde Stuttgart, Germany (SMNS). Two samples labelled as MP031 (female) and MP032 (male) will be preserved at FH (Farlow Herbarium, Harvard University, Cambridge, Massachusetts) and the Muséum national d’Histoire naturelle, Paris, France (MNHN), respectively. No details are available about amber deposits or collecting conditions. The newly described fossil species of Laboulbeniales, †*Columnomycetes electri*, is attached to the right metatibia of specimen MP031 (Fig. 1a).

Preliminary screening of specimens of *Proptomaphaginus hispaniolensis* and *Proptomaphaginus puertoricensis* in the personal collection of M. Perreau in Paris, France (CMPR) resulted in the discovery of Laboulbeniales thalli on both species. We reached out to Dr. Stewart S. Peck, whose insect collection is preserved at the Canadian Museum of Nature (CMN) in Ottawa. His screening efforts revealed more infected specimens of *Proptomaphaginus*, which were kindly sent to the second author for study of their associated parasites.

External and internal structures of the Coleoptera were illustrated using both visible light observations and propagation phase-contrast synchrotron X-ray microtomography (PPC-SRμCT)^28^.

### Propagation phase contrast synchrotron X-ray microtomography

Microtomographic observations allow virtually dissecting specimens in a non-destructive way^29^ and displaying the cellular structure of associated organisms, such as ectoparasitic Laboulbeniales fungi. PPC-SRμCT was performed at the European Synchrotron Radiation Facility (ESRF, Grenoble, France). Scans were performed on the beamline ID19 (for MP031, MP032, MP033) or BM5 (MP025) with a monochromatic X-ray beam at the energy of 20 keV, using a multilayer monochromator. The CCD detector was a FreLoN HD2k (fast read-out low noise) with 2048 × 2048 pixels, coupled to a microscope system with a single crystal YAG:Ce scintillator screen of 25 mm of thickness. The resolution (voxel size) of the scans of MP025, MP031, and MP032 was 0.685 μm. Two pixel sizes were used for MP033, 1.406 μm and 0.703 μm. A continuous rotation was used to blur out undesired details located outside the field of interest (far from the rotation center) to decrease their contribution to the noise of the final reconstructed slices^30^. A special scan was performed on MP031 to resolve the parasitic fungus at 0.212 μm, with a distance sample-detector of 20 mm and with 1500 projections over 180°, also with a continuous rotation. In-house software packages present at ESRF were used for tomographic reconstructions. Segmentations were done with Vgstudiomax 2.1 (Volumegraphics, Heidelberg, Germany) on a computer based on an AMD motherboard Magny-Cours (48 cores) and 512 Go of random-access memory.

**Figure 2:**
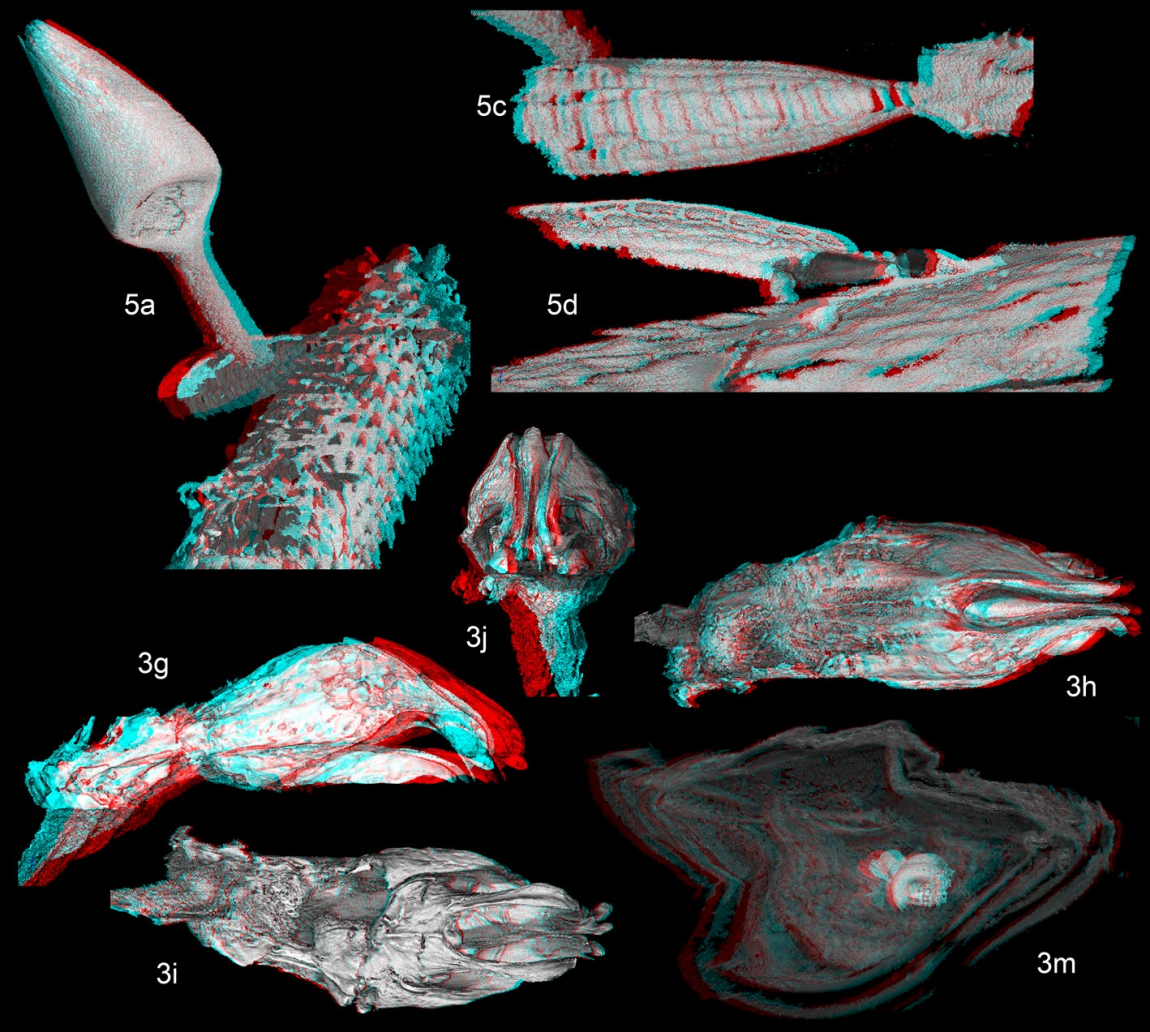
Anaglyph pictures of 2g–2j; 2m; 4a; 4c; 4d. †*Proptomaphaginus alleni* and †*Columnomyces electri*, pictures in anaglyph red/cyan of three-dimensional view of selected morphological details. The numbering is the same as the corresponding flat pictures.

In addition to the standard pictures, selected figures are also presented in anaglyph red/cyan to visualize the three-dimensional structures of key morphological details with red/cyan-filtered glasses. They are presented in Fig. 2; numbering is the same as the corresponding flat figures.

### Modern specimens imaging

**Figure 3:**
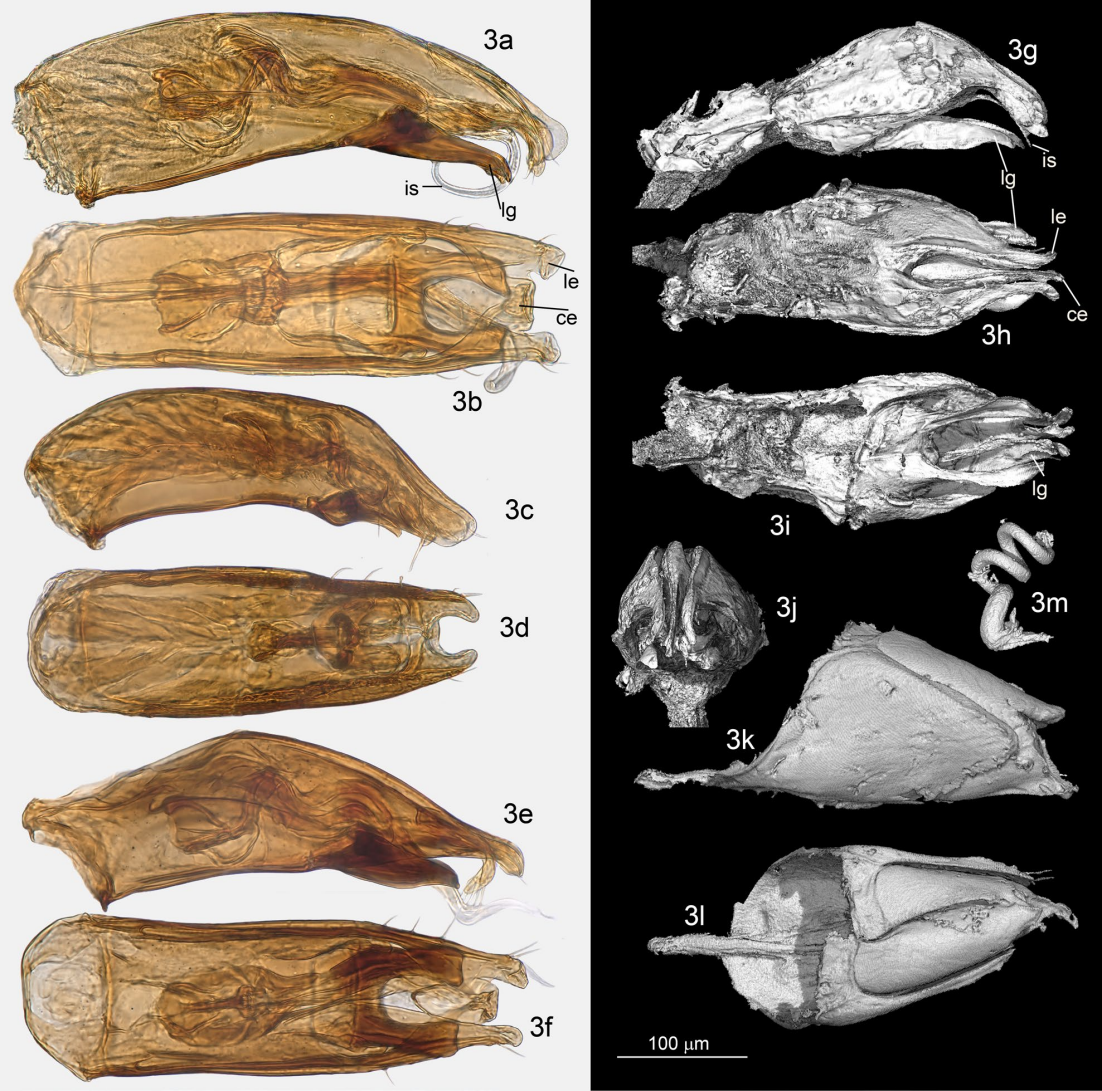
Genus *Proptomaphaginus*, genital structures. (**a**) *P. hispaniolensis*, aedeagus left lateral view. (**b**) *P. hispaniolensis*, aedeagus dorsal view. (**c**) *P. puertoricensis*, aedeagus left lateral view. (**d**) *P. puertoricensis*, aedeagus dorsal view. (**e**) *P. apodemus*, aedeagus, left lateral view. (**f**) *P. apodemus*, aedeagus, dorsal view. (**g**) †*P. alleni*, aedeagus left lateral side. (**h**) †*P. alleni*, aedeagus dorsal view. (**i**) †*P. alleni*, aedeagus ventral view. (**j**) †*P. alleni*, aedeagus frontal view. (**k**) †*P. alleni*, male genital segment lateral view. (**l**) †*P. alleni*, male genital segment dorsal view. (**m**) †*P. alleni* (specimen MP033), wide base of spermaduct. Abbreviations: ce = central expansion, is = internal stylus, le = lateral expansion, lg = ligulae.

**Figure 4:**
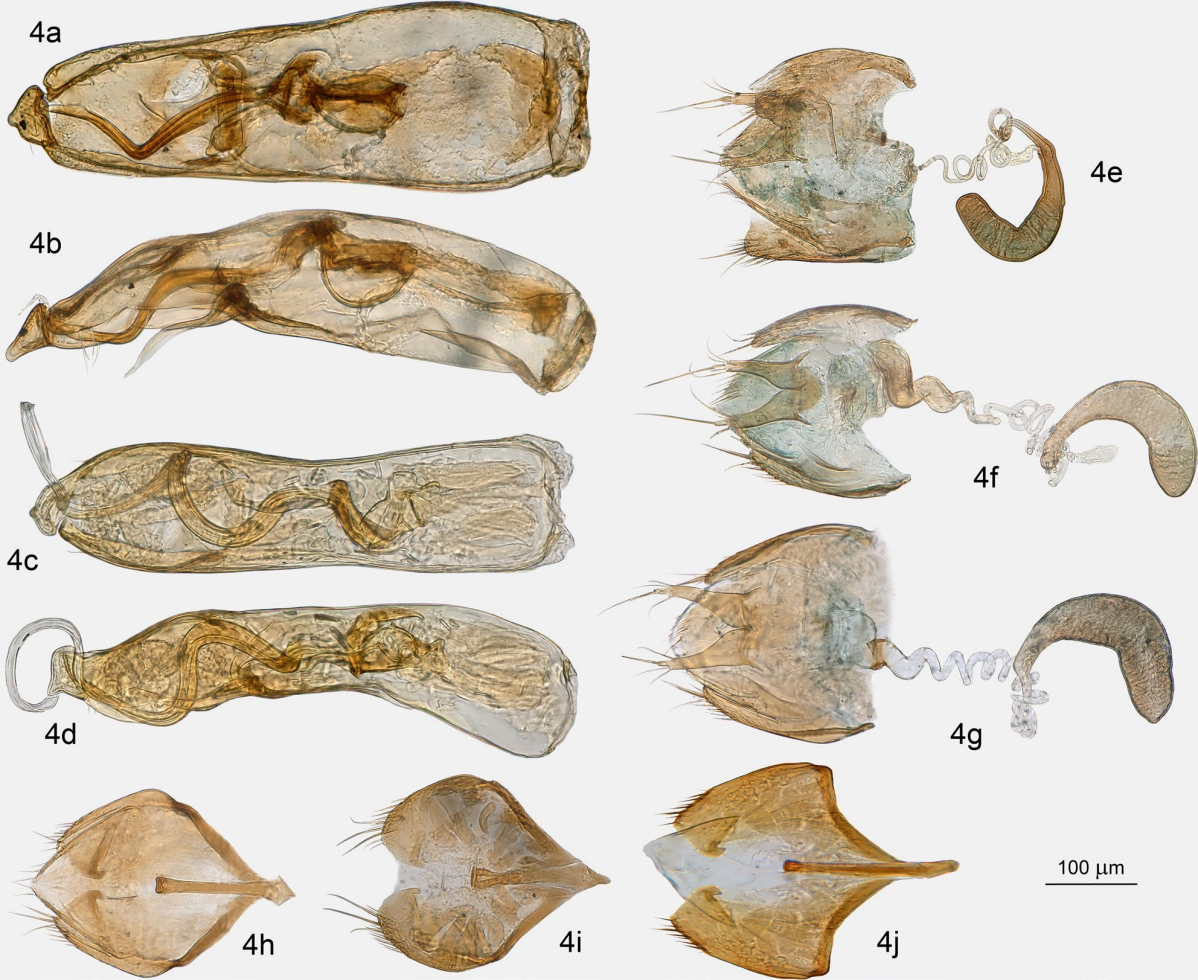
Genus *Proptomaphaginus*, genital structures. (**a**) *P. microps*, aedeagus dorsal view. (**b**) *P. microps*, aedeagus lateral view. (**c**) *P. reddelli*, aedeagus dorsal view. (**d**) *P. reddelli*, aedeagus lateral view. (**e**) *P. puertoricensis*, female genital segment. (**f**) *P. hispaniolensis*, female genital segment. (**g**) *P. apodemus*, female genital segment. (**h**) *P. apodemus*, male genital segment. (**i**) *P. puertoricensis*, male genital segment. (**j**) *P. hispaniolensis*, male genital segment.

After dissection, genital structures of Coleoptera were treated as follows. The aedeagus of male specimens was dehydrated in 95% ethanol, before being mounted in Euparal on a microscope slide. Female genitalia were cleared in hot 10% KOH for 10 min, stained with a diluted ethanolic solution of Azoblack^31^, rinsed in demineralized water, and then mounted in dimethyl-hydantoin-formaldehyde (DMHF) on a microscope slide. Visible light photographs were captured by a Spot Insight IN1820 camera attached on a Leica M10 stereomicroscope for Fig. 1a, and by a Keyence VHX5000 microscope with lens VH-Z250T for Figs. 3a–f, 4a–j.

Screening for Laboulbeniales was done under 40–50 × magnification. Thalli of Laboulbeniales were carefully removed at the foot using a Minuten Pin (BioQuip #1208SA, Rancho Dominguez, California) of which the tip was dipped in Hoyer’s medium to prevent thalli from getting lost during transfer to microscope slide. Mounting of specimens in Amann’s medium was done applying a double coverslip technique using Solakryl BMX (ENTO SPHINX s.r.o., Pardubice, Czech Republic)^32^. Photomicrographs were taken with an Olympus SC30 camera mounted on an Olympus BH2 bright field compound microscope and viewed and processed using cellSens 1.18 imaging software (Olympus, Tokyo, Japan). Line and stipple drawings were made with PITT artist pens (Faber–Castell, Nürnberg, Germany) based on photomicrographs. All slides are deposited at the Kriebel Herbarium, Purdue University (PUL; West Lafayette, Indiana). Herbarium acronyms are according to Index Herbariorum^33^.

### Parsimony phylogenetic analysis of *Proptomaphaginus*

We performed the phylogenetic analysis of the genus *Proptomaphaginus* using a matrix comprising nine terminal taxa, with *Ptomaphagus* as out group and 23 characters of which 17 were parsimony-informative, 7 were external characters, 12 were characters of male genitalia; and 4 were characters of female genitalia.

The reduction of eyes was not included since this is generally associated with an hypogean adaptation, frequent in Ptomaphagini, and is likely homoplasic. The sexual dimorphism of the apex of elytra (Figs. 1d,e) was also excluded because this is a highly variable character in the genera *Ptomaphagus*, *Ptomaphaginus*, and *Ptomaphaminus*.

The matrix was compiled using Paup* 4.0b10^34^. A first analysis was done by exhaustive search of maximum parsimony. All characters were equally weighted and multi-state characters were treated as unordered. Next, a bootstrap analysis based on a heuristic search with 10,000 replicates was performed. Tree visualization and mapping of characters were done in Winclada 1.00.08^35^.

## Results

### Systematic paleontology of the host

Phylum **Arthropoda** von Siebold, 1848.

Classis **Insecta** Linnaeus, 1758.

Ordo **Coleoptera** Linnaeus, 1758.

Superfamilia **Staphylinoidea** Latreille, 1802.

Familia **Leiodidae** Fleming, 1821.

Subfamilia **Cholevinae** Kirby, 1837.

Tribus **Ptomaphagini** Jeannel, 1911.

Subtribus **Ptomaphaginina** Szymczakowski, 1964.

Genus ***Proptomaphaginus*** Szymczakowski, 1969.

**†*Proptomaphaginus alleni*** **Perreau & Haelewaters, sp. nov.**

urn:lsid:zoobank.org:act:0D9286F4-F5BB-4B98-8C19-DC32774CE522.

#### Types

Holotype: **Dominican Republic**, male specimen, Dominican amber, MP032 (MNHN: MNHN.F.A.71339). Paratypes: **Dominican Republic**, Dominican amber, male specimen, MP025 (SMNS: DO1468K); female specimen, MP031 (FH); female specimen, MP033 (SMNS: DO489K).

#### Etymology

Named in recognition of Albert D. Allen, a private beetle enthusiast, who provided the first specimens of this fossil species.

#### Description

*Habitus*: Fig. 1a,b. *Head* with normally developed eyes (Fig. 1f), and with an occipital carina. Punctation aligned in transverse microstrigae. Antennae compact, continuously widened from base to apex, the eighth antennomere strongly transverse (Fig. 1c). *Pronotum* approximately 1.3 × longer than wide, the widest at base, with dorsal punctation aligned in transverse microstrigae. *Elytra* elongated, approximately 1.7 × longer than combined width, with a single parasutural longitudinal stria. Apex sexually dimorphic, widely rounded in female (Fig. 1d), truncate in males (Fig. 1e). Flight wings present. *Ventral structures*: mesoventral process low, narrow and rounded, with some setae on the ventral surface. Epimeron of the mesoventrum transverse. Metaventrum prominent longitudinally on the median line, with a longitudinal median groove. Metaventrahl lateral sutures roughly parallel to the body axis (“ms” in Fig. 1f). Abdomen with six visible segments. Legs: protibiae with a single row of regular spines along the external edge (Fig. 1g), ventral spines are randomly distributed on the ventral surface. Mesotibiae and metatibiae with a comb of regular spines surrounding the insertion of tarsi. Male protarsi weakly dilated (Fig. 1g), female protarsi undilated. *Male genital segment* with a long and narrow *spiculum gastrale*, protruding forward beyond the anterior edges of the epipleurites by half of its length. Epipleurites with setae along the posterior edge (Fig. 3k,l). Aedeagus (Fig. 3g–j) fairly thick, median lobe with two lateral apical prominent expansions (“le” in Fig. 3h) and one central less prominent expansion (“ce” in Fig. 3h). Lateral sides narrowed at apex in dorsal view (Fig. 3h). Ventral ligulae strongly developed (“lg” in Fig. 3g–i). The internal stylus partly protruding beyond the ventral orifice of the median lobe (“is” in Fig. 3g). Lateral expansions and stylus are also clearly visible in frontal view (Fig. 3j). At least two lateral setae and one apical seta are present. The median lobe was perhaps compressed laterally in its apical part during diagenesis since the ventral ligulae which generally closes the genital orifice significantly oversteps laterally the lateral margins of the median lobe. Female genital segment indistinct in the two female paratypes, but a helical spermaduct with a wide base is partly visible in specimen MP033 (Fig. 3m). The apical capsule that generally has the shape of a club in *Proptomaphaginus* is missing, likely resulting from a preservation artifact. *Length* 2.0–2.3 mm (approximated from the sum of lengths of head, pronotum, and elytra from the scutellum).

#### Notes

The external morphology is extremely similar to the single known extant species from the island of Hispaniola (Haiti and the Dominican Republic): *Proptomaphaginus hispaniolensis*. Eyes are normally developed, differing from other extant species that have reduced eyes as an adaptation to a hypogean lifestyle^26^. No reliable external characters are available. We eventually detected differences in the size of antennomeres but cannot exclude the possibility that these are preservation artifacts. However, male genital structures, which are important for species identifications of Leiodidae, give clear characters to distinguish the fossil species from its extant Caribbean relatives. In dorsal view, the apical part of the aedeagus—the only part that is well preserved—is much more narrowed, with the two lateral expansions of the median lobe nearly contiguous (“le” in Fig. 3h) while they are distant in the extant Caribbean species, *Proptomaphaginus hispaniolensis* (Fig. 3b), *Proptomaphaginus puertoricensis* (Fig. 3d), and *Proptomaphaginus apodemus* (Fig. 3f). In lateral view, lateral expansions are more sinuous and more prominent (Fig. 3g *versus* Fig. 3a,c,e). The internal stylus of the endophallus (“is” in Fig. 3a,g), which is partly protruding outside the median lobe at the apex in *Proptomaphaginus hispaniolensis* is also visible in †*Proptomaphaginus alleni* but seems straight rather than curved. The male genital segment is similar in *Proptomaphaginus hispaniolensis* (Fig. 3j) and †*Proptomaphaginus alleni* (Fig. 3l), with a very long, thin *spiculum gastrale* protruding beyond the anterior margin of the epipleurites.

### Systematic paleontology of the parasite

Phylum **Ascomycota** Caval.-Sm.

Classis **Laboulbeniomycetes** Engl.

Ordo **Laboulbeniales** Lindau.

Familia **Laboulbeniaceae** G. Winter.

Tribus **Laboulbenieae** Thaxt.

Subtribus **Laboulbeniinae** I.I. Tav.

Genus ***Columnomyces*** R.K. Benj.

†***Columnomyces electri*** **Haelew. & Perreau, sp. nov.**

MycoBank MB 830254.

**Figure 5:**
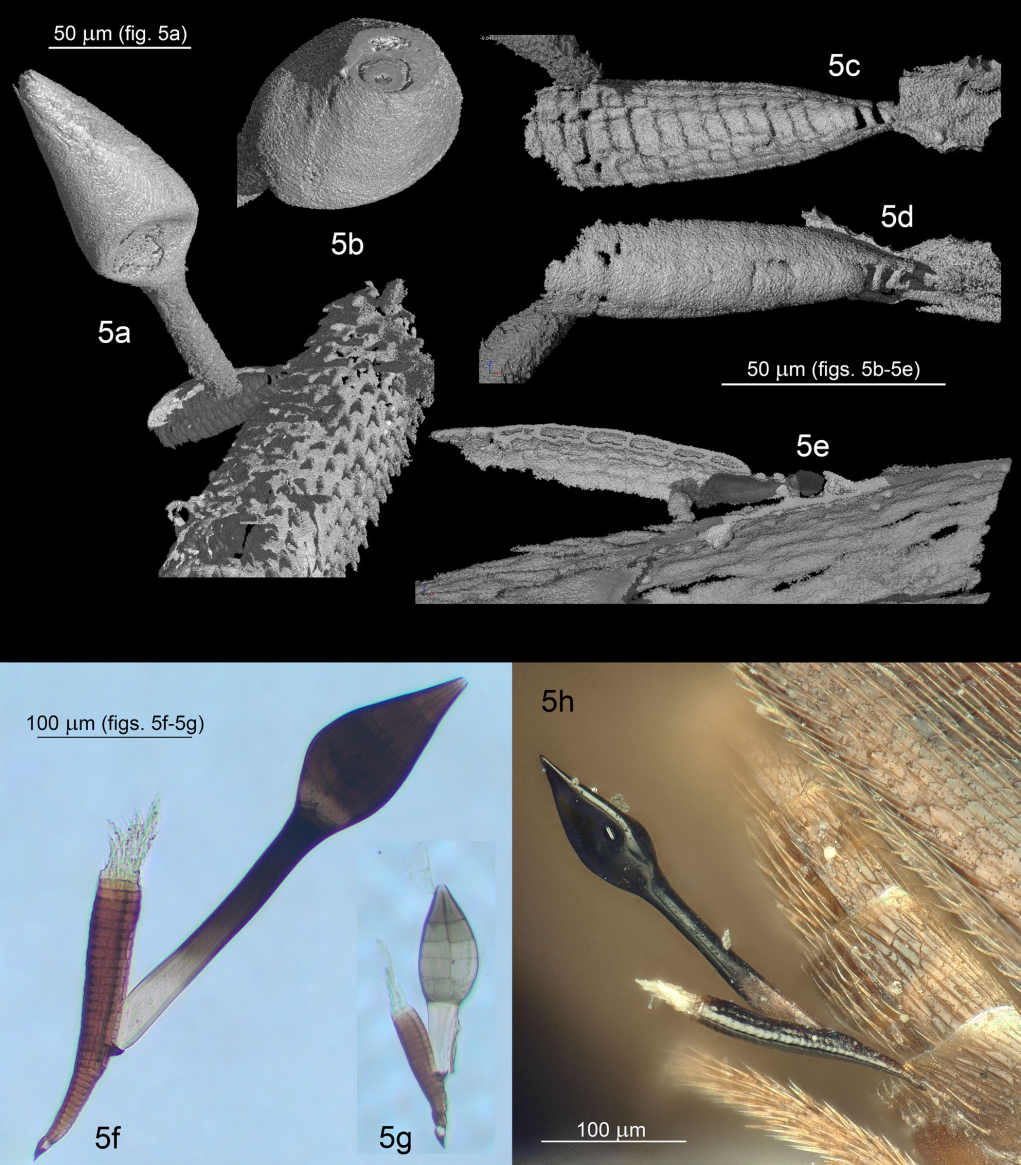
Genus *Columnomyces*. (**a**) †*C. electri* sp. nov., mature thallus. (**b**) †*C. electri*, perithecium, with transversal cut at the apex showing the two apical cells. (**c**) †*C. electri*, receptacle, internal side. (**d**) †*C. electri*, receptacle, external side. (**e**) †*C. electri*, receptacle transversal cut. (**f**) *C. peckii* sp. nov., mature thallus. (**g**) *C. hispaniolensis* sp. nov., mature thallus. (**h**) *C. peckii*, mature thallus attached to its host’s sternite (*in-situ*).

**Figure 6:**
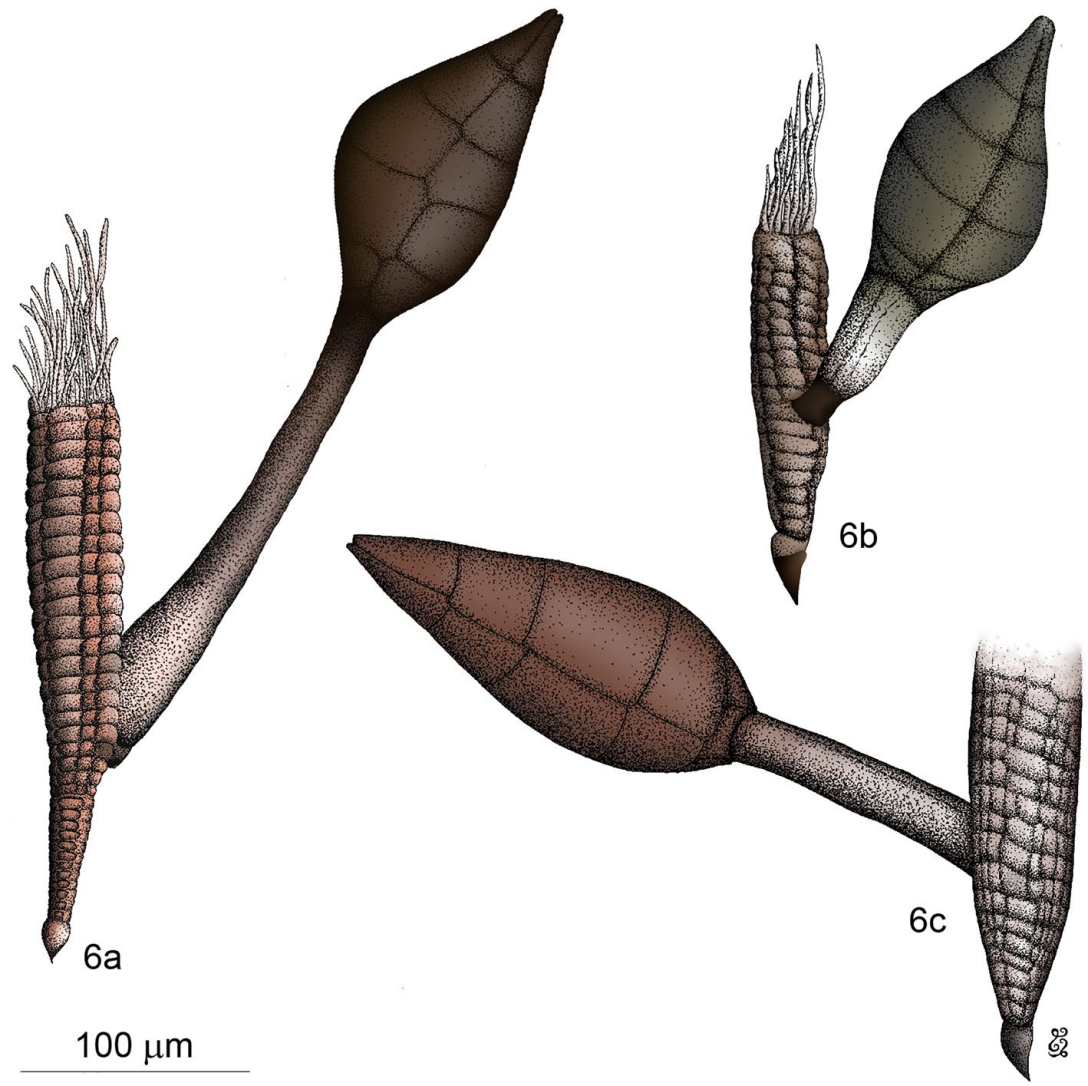
Genus *Columnomyces*. (**a**) *C. peckii* sp. nov., mature thallus. (**b**) *C. hispaniolensis* sp. nov., mature thallus. (**c**) †*C. electri* sp. nov., mature thallus, artist’s interpretation. Scale bar: 5a = 100 μm: b–5c = 50 μm. Drawings by Jingyu Liu.

Figures 5a–e, 6c; Supplementary File 1, Supplementary Video 1.

#### Type

Holotype: **Dominican Republic**, Dominican amber, 1 mature thallus on right metatibia of †*Proptomaphaginus alleni* sp. nov., MP031, D. Haelew. 211 (FH).

#### Etymology

Latin for "from amber".

#### Description

*Receptacle* 77 × 27 µm, multicellular, semicircular in cross section; cells above the two single basal-most cells compacted to a pseudoparenchyma, consisting of at least eight superposed tiers of cells, cells from the third tier onwards arranged in 2–3 rows; the median row consisting of rectangular to flattened cells; the lateral rows consisting of cells alternating with those in the median row, each cell longer than wide, sometimes divided into two. *Cell VI* 68 × 13 µm, slender, elongated, with parallel margins; detached from the receptacle likely as a result of preservation. *Perithecium* 110 × 41 µm, asymmetrical, obclavate, broadest at its lower third, one margin much more convex than the other in longitudinal section; bearing two minute teeth on opposite sides around the septum between tiers III and IV of perithecial wall cells; venter without transition tapering upwards; apex undifferentiated, consisting of two unequal lips, one of which is large and rounded, the other small and narrow.

### Taxonomy of the extant fungal parasites

***Columnomyces hispaniolensis* Haelew. & Perreau sp. nov.**

MycoBank MB 835552.

Figures 5g, 6b.

#### Types

Holotype: **Dominican Republic**, La Romana Province, cave at mouth of Rio Chavón, 8 May 1978, *leg.* R.E. Woodruff & G.B. Fairchild, black light, on *Proptomaphaginus hispaniolensis* Peck, 1983 (Coleoptera, Leiodidae, Cholevinae, Ptomaphagini, Ptomaphaginina) (Canadian Museum of Nature, CMN), slide D. Haelew. 1627b (PUL F27079, 1 mature thallus, left metatibia). Isotypes: *Ibid.*, slides D. Haelew. 1627a (PUL F27078, 1 mature thallus, sternite) and D. Haelew. 1627c (PUL F27080, 2 mature thalli, right elytron). Paratypes: *Ibid.*, on *Proptomaphaginus hispaniolensis* (CMN), slide D. Haelew. 1628a (PUL F27080, 1 submature thallus, left margin of pronotum). La Vega Province, Parque Nacional José Armando Bermúdez, La Cienaga, 1100 m a.s.l., 19 July–2 August 1995, *leg.* S.B. Peck & J. Peck, on *Proptomaphaginus hispaniolensis* (personal collection of M. Perreau, CMPR), slide D. Haelew. 1509a (PUL F27669, many juvenile thalli, right elytron). *Ibid.*, on *Proptomaphaginus hispaniolensis* (CMPR), slide D. Haelew. 1510a (PUL F27670, 1 submature thallus, right metatarsus). *Ibid.*, on *Proptomaphaginus hispaniolensis* (CMPR), slide D. Haelew. 1511a (PUL F27671, 1 mature thallus with perithecium broken off, right metatarsus). **Haiti**, Département du Sud, 1 mi SSW Camp-Perrin, Grotte de Counoubois, 2 November 1979, *leg.* J.M. Stock, J.R. Holsinger, et al*.*, on *Proptomaphaginus hispaniolensis* (CMN), slide D. Haelew. 1629a (PUL F27082, 1 mature thallus, tergite).

#### Etymology

Referring to the host species, *Proptomaphaginus hispaniolensis*, to emphasize the presumed strict host specificity and biogeographical patterns that have resulted in these intimate parasite–host associations.

#### Description

*Thallus* 108–147 µm long from foot to tip of appendages, 170–248 µm long from foot to perithecial apex; receptacle colored rust-brown; basal cell above the blackened foot, cell VI, and appendages hyaline; perithecium ash gray to brown, darkening with age; in one older thallus, the upper quarter of cell VI is also darkened. *Receptacle* 66–102 × 19–28 µm, multicellular, semicircular in cross section, consisting of 17–22 superposed tiers of flattened cells (except those in the upper 2–3 tiers); 5–8 lowest tiers each consisting of a single cell, broadening upwards; cells above the 5–8 basal-most ones arranged in 2–3 rows; upper 2(–3) tiers narrower than those below, more-celled, with cells longer than wide. *Appendages* many, arising from upper tiers of receptacular cells, septate, slender, reaching up to 52 × 1.4–2.0 µm; some antheridial, some sterile. *Cell VI* 49–105 × 20–28 μm, between 2.5 and 4.1 × longer than wide, trapezoidal, slightly to conspicuously broadening upwards; arising laterally from one cell of a tier (7 to 10), attached to it with a darkened, obtriangular-shaped, foot-like structure. *Perithecium* 73–113 × 48–61 μm, between 1.4 and 2.0 × longer than wide, ovoid, broadest at lower third, symmetrical or more-often asymmetrical, with anterior margin more convex than posterior margin; upper part tapering; apex smooth and blunt, undifferentiated, symmetrical.

***Columnomyces peckii *Haelew. & Perreau, sp. nov.**

MycoBank MB835553.

Figures 5f,h, 6a.

#### Types

Holotype: **Puerto Rico**, Aguas Buenas, Aguas Buenas Cave [Reserva Natural Sistema de Cuevas y Cavernas de Aguas Buenas], 250 m a.s.l., 17 May 1973, *leg.* S. Peck et al*.*, on *Proptomaphaginus puertoricensis* Peck, 1970 (Coleoptera, Leiodidae, Cholevinae, Ptomaphagini, Ptomaphaginina) (MNHN), slide D. Haelew. 1486a (PUL F27072, 1 mature thallus, sternite). Paratypes: *Ibid.*, on *Proptomaphaginus puertoricensis* (CMPR), slide 1485a (PUL F27071, 1 juvenile and 1 submature thallus, right metatibia). *Ibid.*, on *Proptomaphaginus puertoricensis* (CMPR), slide 1508a (PUL F27073, 1 juvenile thallus, right elytron). *Ibid.*, on *Proptomaphaginus puertoricensis* (CMN), slide D. Haelew. 1595a (PUL F27074, 1 submature and 1 mature thallus, junction of left metatibia and -tarsus). *Ibid.*, on *Proptomaphaginus puertoricensis* (CMN), slide D. Haelew. 1624a (PUL F27075, 3 thalli, left metatibia). *Ibid.*, on *Proptomaphaginus puertoricensis* (CMN), slide D. Haelew. 1625a (PUL F27076, 8 thalli of which 2 mature and with complete perithecium, elytra). **Puerto Rico**, Isabela Municipio, Barrio Mora, Cueva de los Alferos, 4 June 1958, leg. M.W. Sanderson, in bat guano, on *Proptomaphaginus puertoricensis* (CMN), slide D. Haelew. 1626a (PUL F27077, 1 immature thallus, right mesotibia).

#### Etymology

In recognition of Stewart B. Peck, Research Associate at the Canadian Museum of Nature (Ottawa, Canada), as a tribute to his enormous contributions to the knowledge of New World Leiodidae and subterranean Coleoptera, as well as his special interest in the Caribbean fauna and its biogeography.

#### Description

*Thallus* 254 µm long from foot to tip of appendages, 233–422 µm long from foot to perithecial apex; colored amber-brown; basal cell of the receptacle above the blackened foot, the appendages, and the lower quarter to (maximum) half of cell VI hyaline; the remaining part of cell VI and perithecial venter darkening with age. *Receptacle* 113–192 × 25–30 µm, multicellular, semicircular in cross section, consisting of 18–36 superposed tiers of flattened cells (except those in the upper 2–3 tiers); 5–13 lowest tiers each consisting of a single cell, broadening upwards; cells above the 5–13 basal-most ones arranged in 2–3 rows; upper 2–3 tiers many-celled, with cells longer than wide. *Appendages* many, arising by proliferation from upper 3–4 tiers of receptacular cells, septate, slender, reaching up to 56 × 1.5–3.0 µm; some antheridial, some sterile. *Cell VI* 69–203 × 19–21 μm, between 3.6 and 9.7 × longer than wide, slender, with parallel margins, straight; arising laterally from one cell of a tier (10 to 17), attached to it with a darkened, obtriangular-shaped, foot-like structure. *Perithecium* 104–136 × 50–63 μm, 2.1 × longer than wide, ovoid to pyriform, broadest at lower third, asymmetrical, with one margin more convex than the other; upper part tapering; apex smooth and blunt, undifferentiated, symmetrical.

#### Notes

The three extant species of *Columnomyces* can be distinguished based on morphological evidence. *Columnomyces peckii* stands out because of its size (Fig. 5f,h); it is generally conspicuously larger compared to the other species. Compared to *Columnomyces hispaniolensis*, the perithecium of *Columnomyces peckii* is similar in width but it is generally longer; this is reflected in the length/width ratios: 1.4–2.0 for *Columnomyces hispaniolensis*, 2.1 for *Columnomyces peckii*. Compared to *Columnomyces ptomaphagi*, the receptacular tiers, cell VI, and the perithecium of *Columnomyces peckii* are noticeably narrower, resulting in *Columnomyces peckii* having a slenderer habitus. Finally, while several measurements and ratios partly overlap between these two species, *Columnomyces ptomaphagi* can still be easily distinguished from *Columnomyces hispaniolensis* based on the width of the receptacular tiers (35–40 versus 19–28 μm in *Columnomyces hispaniolensis*), the number of tiers consisting of a single cell (2–3 versus 5–8 in *Columnomyces hispaniolensis*), and cell VI, which is both longer and wider. An overview of morphological comparisons among all four species of *Columnomyces *is presented in Table 1.

**Table 1:** Comparative table of descriptive characteristics for currently described species of *Columnomyces*. All measurements are in μm.

	†*C. electri*	*C. hispaniolensis*	*C. peckii*	*C. ptomaphagi*
Total length	N/A	170–248	233–422	230
**Receptacle**				
Measurements	77 × 27	66–102 × 19–28	113–192 × 25–30	120 × 35–40
Number of tiers	At least 8	17–22	18–36	Up to 18
Tiers with single cell	2	5–8	5–13	2–3
Cells lateral rows	Longer than wide	Flattened	Flattened	Flattened
**Cell VI**				
Measurements	68 × 13	49–105 × 20–28	69–203 × 19–21	120 × 30
L/W ratio	5.2	2.5–4.1	3.6–9.7	3.7
**Perithecium**				
Measurements	110 × 41	73–113 × 48–61	104–136 × 50–63	95 × 65
L/W ratio	2.7	1.4–2.0	2.1	1.5
Minute teeth	2 on opposite sides	Absent	Absent	Absent
**Reference**	This paper	This paper	This paper	Benjamin (1955)

Due to diagenesis (see “Discussion”), appendages and antheridia are missing in the amber-embedded thallus of †*Columnomyces electri*. It seems that (at least) the upper three of four tiers of the receptacle are missing. In addition, cell VI is detached from the receptacle. Despite these damages, we were able to assign the preserved thallus to the genus *Columnomyces* based on the pseudoparenchymatous receptable. In addition, †*Columnomyces electri* can be easily separated from the other species in the genus by several features: the cells of the receptacular lateral rows are longer than wide, while they are flattened in the other species; cell VI is much narrower; the perithecium of †*Columnomyces electri* is much more elongate, with the length/width ratio (2.7) far exceeding that of the other described species; and two minute teeth are present at the height of the septum between the two upper tiers of perithecial wall calls (Supplementary Video 1).

### Host phylogeny

The first morphology-based phylogeny of the extant species of *Proptomaphaginus* was presented by Peck^26^. We extended his analysis to include the newly described fossil species and the two most species-rich Asian genera that belong to the same subtribe of Ptomaphagini, Ptomaphaginina. These genera are *Ptomaphaginus* and *Ptomaphaminus*. *Ptomaphagus*, which belongs to another subtribe of Ptomaphagini (Ptomaphagina), was selected as outgroup. The phylogeny does not aim to resolve the relationships among all Ptomaphagini or Ptomphaginina, rather it aims to make more precise the placement of *Proptomaphaginus*. We used 23 characters, of which 17 were parsimony-informative. The list of characters and the corresponding data matrix are given in Supplementary File 2.

**Figure 7:**
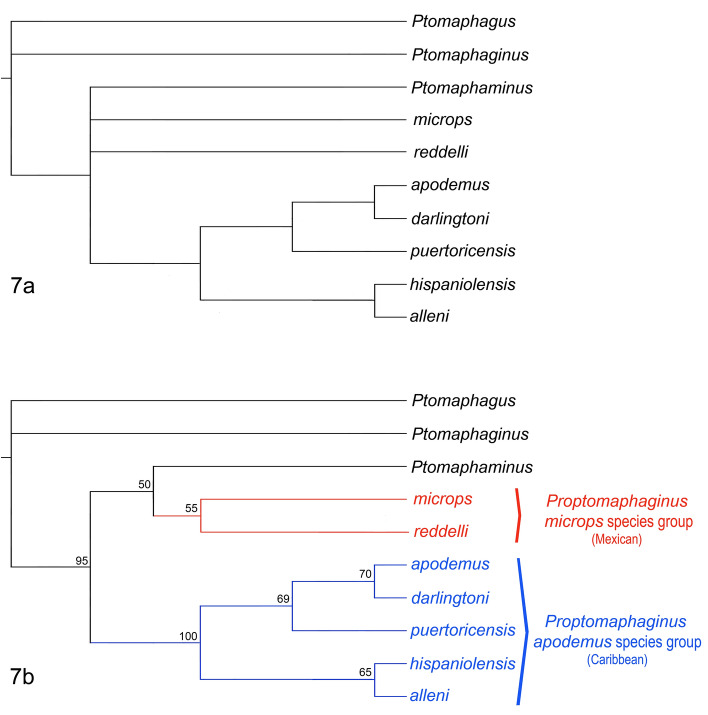
Phylogeny of the genus *Proptomaphaginus*. (**a**) Strict consensus tree of the 3 most parsimonious trees obtained from an exhaustive search based on our character matrix. (**b**) Tree obtained after a heuristic search with 10,000 replicates. Bootstrap support (if ≥ 50) indicated at each node*.*

An exhaustive search resulted in three minimal trees. The strict consensus tree is shown in Fig. 7a. A bootstrap analysis based on a heuristic search with 10,000 replicates resulted in the tree shown in Fig. 7b.

Relationships among *Proptomaphaginus microps*, and *Proptomaphaginus reddelli*, and the Asian genus *Ptomaphaminus* are unresolved, resulting in a polytomy. The monophyly of the set of Caribbean species received maximum bootstrap. This suggests that Mexican species and Caribbean species may belong to two different genera. The corresponding apomorphies are (*i*) the thick ventral ligulae closing the apical orifice of the median lobe (*versus* thin ligulae for Mexican species and other genera of Ptomaphaginina), (*ii*) the presence of three apical expansions to the median lobe of the aedeagus (*versus* only two for Mexican species and other genera of Ptomaphaginina), (*iii*) the approximately symmetric shape of the aedeagus, (*iv*) the repartition of the lateral setae of the median lobe of the aedeagus, and (*v*) the presence of apical setae at the tip of the median lobe of the aedeagus.

The sister relationship of *Proptomaphaginus apodemus* and *Proptomaphaginus darlingtoni* is also supported (bootstrap 70%). Their identity as two different species has been extensively discussed, before Peck^36^ gave a characterization based on genital characters. The two Hispaniolan species †*Proptomaphaginus alleni* and *Proptomaphaginus hispaniolensis* are supported as sister species with a bootstrap of 65%. This clade is retrieved as sister to (*Proptomaphaginus puertoricensis* + (*Proptomaphaginus apodemus* + *Proptomaphaginus darlingtoni*)) with maximum support. Of note is the high support for the sister relationship of (*Ptomaphaminus* + (*Proptomaphaginus microps* + *Proptomaphaginus reddelli*)) and the Caribbean set of *Proptomaphaginus* species (bootstrap 95%). However, as mentioned earlier, the set (*Ptomaphaminus* + (*Proptomaphaginus microps* + *Proptomaphaginus reddelli*) forms an unresolved polytomy. The genus *Ptomaphaminus* is widespread in southeastern Asia, from China to the Sunda Islands^37,38^, whereas *Proptomaphaginus microps* and *Proptomaphaginus reddelli* have a Mexican distribution^26^. We need more characters—sequence data—to resolve this node and to eventually move the Mexican species of *Proptomaphaginus* into a new genus.

## Discussion

### Generic association

Currently four species are recognized in the genus *Columnomyces*—one fossil species and three extant species. The type species of the genus, *Columnomyces ptomaphagi* R.K. Benj., was described from an unidentified species of *Ptomaphagus* (Ptomaphagini, Ptomaphagina) collected at Giant City State Park, Illinois^19^. We were unable to localize the host specimens from the type collection in order to make an identification at species level. However, it seems likely that the host is *Ptomaphagus brevior* Jeannel, 1949. This species has a wide distribution in North America and, more importantly, it is the single species of *Ptomaphagus* that has been reported from Illinois to date^39^. The three other species—†*Columnomyces electri*, *Columnomyces hispaniolensis*, and *Columnomyces peckii*—are associated with three Caribbean species of *Proptomaphaginus* (Ptomaphagini, Ptomaphaginina): †*Columnomyces electri* with †*Proptomaphaginus alleni* in Hispaniola; *Columnomyces hispaniolensis* with the eponymous extant species *Proptomaphaginus hispaniolenis* in Hispaniola; and *Columnomyces peckii* with the extant *Proptomaphaginus puertoricensis* in Puerto Rico. This strict host specificity suggests a long-lasting coevolution between *Columnomyces* and *Proptomaphaginus* since at least the Miocene, which could be extended to the tribe Ptomaphagini if including *Columnomyces ptomaphagi* associated with *Ptomaphagus brevior*.

### Coevolutionary implications

The two extant species of *Columnomyces* are host specific; *Columnomyces hispaniolensis* was found on four specimens of *Proptomaphaginus hispaniolensis*, *Columnomyces peckii* on seven specimens of *Proptomaphaginus puertoricensis*. It is difficult to infer host specificity for both †*Columnomyces electri* (1 thallus available) and *Columnomyces ptomaphagi* (1 juvenile thallus and 1 mature thallus available), due to the lack of material. However, provided the usually strict host specificity in Laboulbeniales fungi^40,41^, we assume that all *Columnomyces* species have a high degree of host specificity. It is interesting that two extant species are found in the Caribbean not only according to their host but also following biogeographical patterns: *Columnomyces hispaniolensis* in Hispaniola and *Columnomyces peckii* in Puerto Rico.

Most thalli of *Columnomyces peckii* are located on legs, some on elytra. Thalli of *Columnomyces hispaniolensis* are located on legs and elytra, but also on the pronotum and abdomen (tergites and ventrites). The single fossil thallus of †*Columnomyces electri* is located on a leg. The type material of *Columnomyces ptomaphagi* was located on the elytra of the host^19^. It seems that *Columnomyces* species do not exhibit any specificity for position on the host.

When available, fossils in the forms of organisms entrapped in soft resin and subsequently preserved for millions of years after amber hardening, are used to describe many organisms around the world as well as to better understand evolutionary relationships among extant species^42^. The state of preservation and completeness of specimens in amber are highly variable among specimens^43^. Damages can occur pre- or perimortem, including loss of appendages while a living organism is struggling to be released from the resin; postmortem during the polymerization of resin, including changes of external morphology and body proportions in soft-bodied organisms such as arthropods and fungi; and post-extraction, either slow or rapid changes outside of the original anoxic sediments, as a result of anthropogenic changes or mechanical treatment^44^.

The single thallus of the fungus †*Columnomyces electri* in the piece of Dominican amber has a dislocated cell VI (a logical consequence of rapid preservation) and it lacks the distal-most tier(s) of the receptacle. This likely represents postmortem distortion—the damages happened during hardening of the amber. As a result of the receptacle missing its upper tier(s) in †*Columnomyces electri*, we do not know for sure how long the receptacle can be, that is, how many tiers of cells it consists of. The uppers tiers also carry the appendages, which are missing in the fossil thallus. In extant species, the appendage system is composed of many slender, septate branchlets up to 50–56 μm in length. Likewise, Benjamin^19^ noted “many broken specimens” among his material of the extant *Columnomyces ptomaphagi*, including the holotype, which also shows a displaced base of cell VI. It was suggested that the pressure of the cover glass might have caused the detachment of cell VI from the rest of thallus, a process that could be somewhat compared to the hardening of amber.

As a final note, previous studies have also observed damage of Laboulbeniales fungi associated with cholevine beetles. This has been linked to the hypogean lifestyle of these hosts^24,45–47^. Almost all cholevine beetles live underground where they feed on all sorts of decaying organic material—litterfall, rotting fungi, dung, carrion, detritus from vertebrate nests, et cetera^48,49^. Given the fact that many thalli of extant species of Laboulbeniales associated with Cholevinae hosts are damaged for myriad reasons, it comes as no surprise that the fossil species, represented by a single thallus, is damaged.

### Biogeography

**Figure 8:**
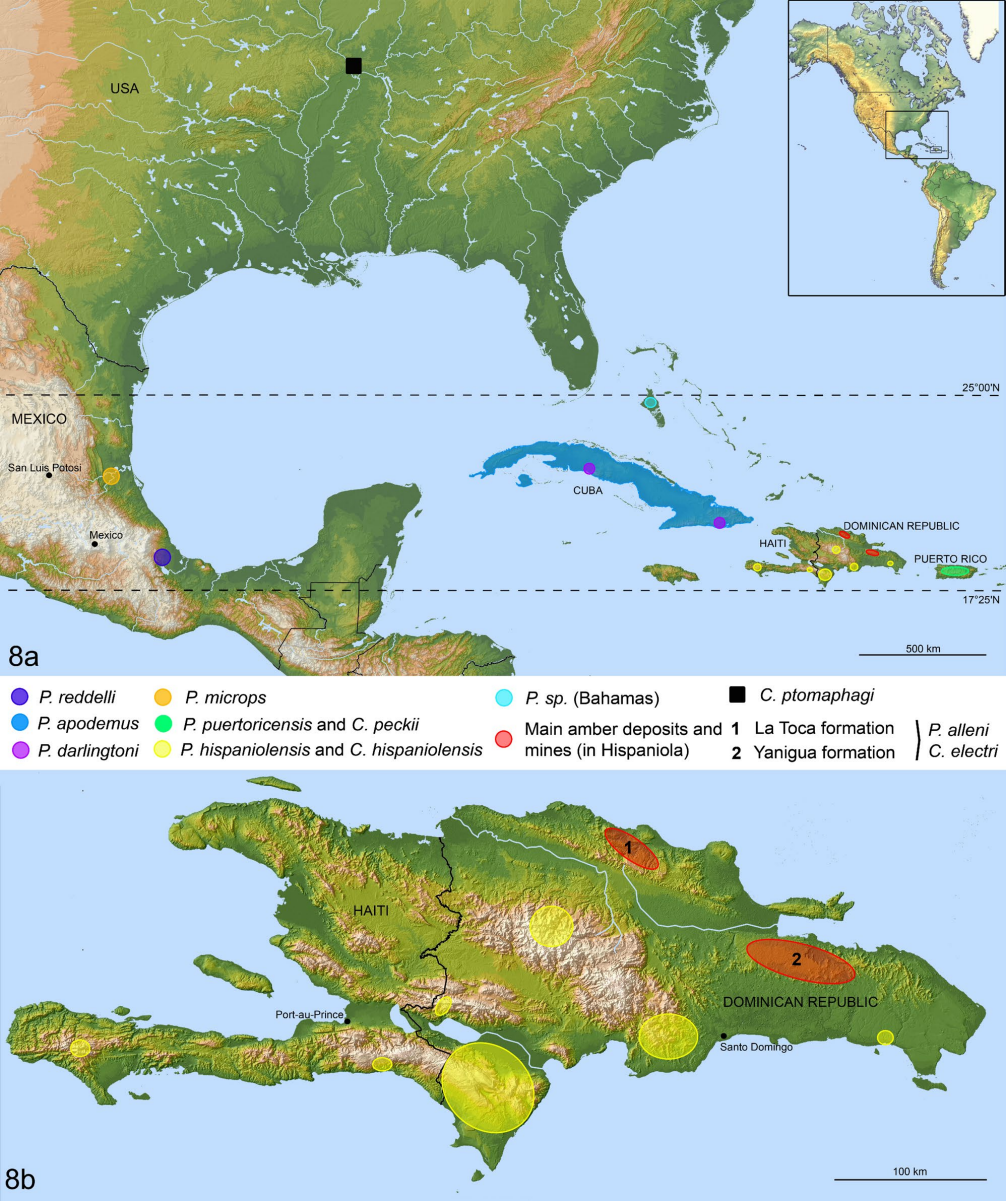
Distribution maps of the genera *Proptomaphaginus* and *Columnomyces*. (**a**) General map. (**b**) Detailed distribution map of *Proptomaphaginus hispaniolensis*, and of main amber deposits and mines in the Hispaniola island, potential locations of †*Proptomaphaginus alleni* with †*Columnomyces electri*. Geographic data sources: ASTER Digital Elevation Model V002; GTOPO30 (USGS^72^); Global administrative area data (https://gadm.org/); Natural Earth data (http://www.naturalearthdata.com/). Data compiled with QGIS 2.18 (https://www.qgis.org).

The distributions of species of the *Proptomaphaginus* species and their *Columnomyces* parasites are illustrated in Fig. 8a,b. Data for extant species of *Proptomaphaginus* are from Peck^26,36,50–52^ and Szymczakowski^53^. Data for extant species of *Columnomyces* are from Benjamin^19^ and this paper. Data on the main amber deposits are from Itturalde Vinent^54,55^.

*Proptomaphaginus* is the only genus of Ptomaphaginina that occurs in tropical America^56^. The other members of the subtribe Ptomaphaginina (*Pandania*, *Ptomaphaginus*, *Ptomaphaminus*) mostly occur in southeastern Asia^38,57,58^.

Peck^56^ suggested that Asian species of Ptomaphaginina derived from ancestors located in North or Central America (proto-Ptomaphaginina sensu Peck^56^). They would have come to occur on the other side of the present Pacific Ocean from Central America (Mexico) in Asia by migration through the Pangea or Laurasia supercontinent before its fragmentation, all European representatives becoming extinct. If true, this would make *Proptomaphaginus* a relict of these ancestors. Under this hypothesis and according to the present phylogeny, their direct descendants are likely members of the “*microps* species group” rather than those of the “*apodemus* species group”. However, we cannot consistently resolve whether the *microps* species group is more closely related to the *apodemus* species group or to the Asian genus *Ptomaphaminus.* The *apodemus* species group is characterized by several autapomorphies that do not occur in any other group of Ptomaphaginina which suggests that it has independently evolved on the Caribbean islands. Unfortunately, no older fossil of Ptomaphaginina is available for assessing this scenario. For example, all fossils of the Cretaceous Albian amber deposit of Kachin in Myanmar (*Cretoptomaphagus microsoma*^59^ and several other undescribed species) belong to Ptomaphagina, not Ptomaphaginina.

## Conclusion

The description of †*Proptomaphaginus alleni* and the discovery and description of the new fossil fungus attached to the right metatibia of one specimen would not have been achieved without the application of **propagation phase-contrast synchrotron X-ray microtomography** (PPC-SRμCT). This technology was introduced to reveal the presence of fossils in fuzzy amber^30^ and has since been applied to make virtual dissections visualizing internal structures at a resolution as high as 0.7 μm^28,29,60–63^. Here, we successfully applied PPC-SRμCT for obtaining a resolution of ~ 0.2 μm. This allowed resolving and understanding cell structures of the fungus, which was necessary for a reliable generic placement. It should be noted that this study was initiated with the discovery of the fossil insect specimens and the associated fossil fungus. Subsequent study of extant species revealed the two new extant species of *Columnomyces*. This is a reversed order of discoveries, compared to the more frequent situation where extant species are described prior to their related fossil congeners.

What contributed to a great extent to this paper was the study of dried, pinned specimens preserved at CMN and CMPR, which revealed two undescribed extant and host-specific species of *Columnomyces*. In total, 115 specimens of *Proptomaphaginus hispaniolensis* were screened, thirteen of which were found with thalli of *Columnomyces hispaniolensis* (parasite prevalence of 11.3%). Similarly, 136 specimens of *Proptomaphaginus puertoricensis* were screened, of which seven were parasitized by *Columnomyces peckii* (5.1%). In recent years, several papers have made a case to increasingly use natural history collections for biodiversity research^6,11,64,65^. Specimens in plant herbaria can be carefully screened for the presence of fungal associates such as downy mildews and rust fungi, leading to identification based on morphology and DNA, descriptions of new species, and studies of host–parasite dynamics^66^. Likewise, **museum insect collections** are being studied for the presence of fungal ectoparasites, resulting in species descriptions^22,67–70^—as also illustrated in our work, studies of host usage patterns^3^, and increased understanding of geographic distributions of the parasites through time^71^.

## Supplementary Information

Supplementary File 1.

Supplementary File 2.

Supplementary Video 1.

## Data Availability

The data concerning the tomographic-reconstructed slices and segmentations are publicly available at the ESRF online paleontological database (http://paleo.esrf.eu).
