# Supplementary material for: A parasitic coevolution since the Miocene revealed by phase-contrast synchrotron X-ray microtomography and the study of natural history collections
Source: Sci Rep. 2021 Jan 29;11:2672. doi: 10.1038/s41598-020-79481-x (PMC7846571; doi:10.1038/s41598-020-79481-x)
Supplement: Supplementary file 1 [file 41598_2020_79481_MOESM1_ESM.pdf]

**A parasitic coevolution since the Miocene revealed by phase-contrast synchrotron Xray microtomography and the study of natural history collections**

Michel Perreau, Danny Haelewaters, Paul Tafforeau

## Supplementary file 1: Description of Supplementary Video 1

Video showing the holotype of †*Columnomyces electri* on the right metatibia of †*Proptomaphaginus alleni.* Reconstructions were made with a single distance phase retrieval using the approach of Paganin[1]. Green parts are regions darker than the amber background, parts in grey are regions lighter than the amber background.

1. Paganin, D., Mayo, S. C., Gureyev, T. E., Miller, P. R. & Wilkins, S. W. Simultaneous phase and amplitude extraction from a single defocused image of a homogeneous object. *J. Microsc.* **206** (1), 33–40 (2002).
