# Supplementary material for: A parasitic coevolution since the Miocene revealed by phase-contrast synchrotron X-ray microtomography and the study of natural history collections
Source: Sci Rep. 2021 Jan 29;11:2672. doi: 10.1038/s41598-020-79481-x (PMC7846571; doi:10.1038/s41598-020-79481-x)
Supplement: Supplementary file 2 [file 41598_2020_79481_MOESM2_ESM.pdf]

A parasitic coevolution since the Miocene revealed by phase-contrast synchrotron X-ray microtomography and the study of natural history collections

Michel Perreau, Danny Haelewaters, Paul Tafforeau

Supplementary file 2: Phylogenetic analysis of the genus *Proptomaphaginus*: Characters list and data matrix.

Characters of external morphology:

1 – Mesepimera: wide (0); narrow (1).

2 – Metaventral suture: parallel to the body axis (0); oblique (1).

3 – Protibiae: roughly cylindrical or conical (0); flat (1).

4 – Arrangement of lateral and apical spines of protibiae: short row of spines of same length along the apical truncation (0); long row of spines of same length along the apical truncation and the lateral external side (1).

5 – Arrangement of ventral spines of protibiae (only for flat protibiae): not relevant (0); randomly spread on the ventral surface (1); aligned along a line parallel to and abut the lateral row (2).

6 – Protarsomeres: distinctly dilated (0); undilated (1).

7 – Mesotarsomere 1: undilated (0); distinctly dilated (1).

Characters of male genitalia:

8 – Male apical orifice of the aedeagus: lateral (0); ventral (1).

9 – Number of apical expansions of the median lobe of the aedeagus: 2 (0); 3 (1).

10 – Ligulae of the median lobe: very thin (Figs. 4b, 4d) (0); uniformly moderately thick (Fig. 3c) (1); very thick and thicker at the base than apex, with regularly decreasing thickness (Figs. 3a, 3g) (2); very thick and abruptly decreasing thickness after the middle (Fig. 3e) (1).

11 – Apex of the median lobe: roughly symmetric in dorsal view (Figs. 3b, 3d, 3f, 3h) (0); asymmetric (Figs. 4a, 4c) (1).

12 – Latero-apical expansions of the median lobe in lateral view: thick and with a more or less straight ventral edge (Fig. 3c) (0); thick with a sinuate ventral edge) (Figs. 3a, 3e, 3g) (1); other conformation (Figs. 4b, 4d) (2).

13 – Central apical expansion of the median lobe in lateral view: not present (0); weakly rolled down (Fig. 3c) (1); strongly rolled down (Figs. 3a, 3e, 3g) (2).

14 – Apex of the median lobe: without setae (Figs. 4b, 4d) (0); with setae (Figs. 3b, 3d, 3f) (1).

15 – Aedeagus in dorsal view: sides parallel or convergent from base to apex (Figs. 3b, 3d, 3f, 3h, 4a) (0); medial constricted (Fig. 4c) (1); preapical constriction (2).

16 – Internal stylus of the endophallus: sinuate (0); helical (1). This character is polymorphic in the genera *Ptomaphaginus* and *Ptomaphaminus*.

17 – Parameres: thin (0); thick (1).

18 – Setae of parameres: grouped at the apex (Figs. 4b, 4d) (0); spread on a short distance before the apex (Figs. 3b, 3d, 3f) (1).

19 – *Spiculum gastrale* of the male genital segment: long and thin, protruding beyond the anterior margin of the epipleurites (Figs. 3l, 4j) (0); long and thin, weakly protruding beyond the anterior margin of the epipleurites and terminated in triangle (Figs. 4h, 4i) (1); short and wide (2).

Characters of female genitalia:

20 – Spermatheca: in shape of a club (Figs. 4e-4g) (0); helical (1). This character is inferred for *P. alleni* to be 0, based on the general shape for all species of Ptomaphaginina.

21 – Spermaduct: sinuate (0); helical (Figs. 3m, 4e-4g) (1). This character is polymorphic in the genera *Ptomaphaminus* and *Ptomaphaginus*.

22 – Spermaduct: without basal sclerite (0); with a basal sclerite (1) (Figs. 4e-4g). Character uncertain for *P. alleni*.

23 – Spermaduct: with an undilated base (Fig. 4e) (0); with a dilated membraneous base (Fig. 4g) (1); with a dilated and sclerotized base (Fig. 4f) (2). This character is uncertain for †*P. alleni* (could be 1 or 2).

Matrix of character states used for the phylogeny of *Proptomaphaginus.*

	1	2	3	4	5	6	7	8	9	10	11	12	13	14	15	16	17	18	19	20	21	22	23
P. apodemus	1	1	1	1	1	1	0	1	1	3	0	1	2	1	0	0	1	1	1	0	1	1	1
P. microps	1	1	1	1	1	1	0	0	0	0	1	2	0	0	0	1	0	0	0	0	0	0	0
P. hispaniolensis	1	1	1	1	1	1	0	1	1	2	0	1	2	1	0	0	1	1	0	0	1	1	2
P. puertoricensis	1	1	1	1	1	1	0	1	1	1	0	0	1	1	0	0	1	1	1	0	1	1	0
P. reddelli	1	1	1	1	1	1	0	0	0	0	1	2	0	0	1	1	0	0	0	0	0	0	0
P. darlingtoni	1	1	1	1	1	1	0	1	1	3	0	1	2	1	2	0	1	1	1	0	1	1	1
†*P. alleni*	1	1	1	1	1	1	0	1	1	2	0	1	2	1	0	0	1	1	0	0	1	?	{12}
Ptomaphaminus	1	1	1	1	1	0	0	0	0	0	1	2	0	0	0	(10)	0	0	0	0	(10)	0	0
Ptomaphaginus	1	0	1	1	2	0	1	0	0	0	1	2	0	1	0	(10)	0	0	2	0	(10)	0	0
Ptomaphagus	*0*	0	0	0	0	0	1	0	0	0	1	2	0	1	0	0	0	0	0	1	0	0	0
